# Problem Behaviours and Relinquishment: Challenges Faced by Clinical Animal Behaviourists When Assessing Fear and Frustration

**DOI:** 10.3390/ani14182718

**Published:** 2024-09-19

**Authors:** Beverley M. Wilson, Carl D. Soulsbury, Daniel S. Mills

**Affiliations:** Animal Behaviour, Cognition and Welfare Group, School of Natural Sciences, University of Lincoln, Lincoln LN6 7DL, UK; csoulsbury@lincoln.ac.uk (C.D.S.); dmills@lincoln.ac.uk (D.S.M.)

**Keywords:** animal behaviour, dogs, emotions, fear, frustration, qualitative

## Abstract

**Simple Summary:**

Problem behaviours are a leading cause of relinquishment of pets. Clinical animal behaviourists (CABs) have to make behavioural assessments and give advice to help owners address problem behaviours. CABs use information presented about a case to make an assessment, which in turn guides their recommendations, but little is known about how CABs make an assessment from the information presented. This study explored this process, particularly in relation to fear and frustration, two emotions that frequently contribute to problem behaviours and can present in superficially similar ways (e.g., as aggression). Interviews with CABs explored their perception of fear and frustration and how they differentiate these in practice. Interviewees generally agreed on the nature of fear; however, there was little consensus about frustration, and many struggled to explain how the two could be differentiated. Typically, personal judgement and intuition were used when making an assessment, with a range of flaws being apparent in their reasoning. This work not only highlights the current inconsistencies in the use of terminology but also issues with the processes used to make an assessment. Therefore, there is clearly a need to develop consensus definitions and a consistent framework for the assessment of emotions in clinical behaviour practice to help pets with problem behaviours.

**Abstract:**

Fear and frustration are two emotions thought to frequently contribute to problem behaviour, often leading to relinquishment. Inferring these emotions is challenging as they may present with some similar general signs, but they potentially require different treatment approaches to efficiently address the behaviour of concern. Although behavioural assessment frameworks have been proposed, it is largely unknown how clinical animal behaviourists (CABs) assimilate information about the emotional state of an animal to inform their behavioural assessment. In other fields (such as both in human and veterinary medicine), the use of intuition and gut feelings, without the concurrent use of an assessment framework, can lead to higher rates of error and misdiagnosis. Therefore, this study used semi-structured interviews of ten CABs and qualitative methods to explore the ways they conceptualise, recognise and differentiate fear and frustration in dogs. Although interviewees perceived fear and frustration as negative affective states that lead to changes in an animal’s behaviour, there was little consensus on the definition or identification or differentiation of these emotions. The use of a scientific approach (i.e., hypothesis-driven and based on falsification of competing hypotheses) for behavioural assessment was highly variable, with individual assessment processes often characterised by tautology, intuition, circular reasoning and confirmation bias. Assessment was typically based on professional judgment, amalgamating information on interpretation of communicative signals, motivation, learning history, breed, genetics and temperament. Given the lack of consensus in the definition of these states, it is clearly important that authors and clinicians define their interpretation of key concepts, such as fear and frustration, when trying to communicate with others.

## 1. Introduction

Every year many pets are relinquished, and problem behaviours have been identified in many countries as the main animal-related factors leading to both the initial relinquishment [1,2,3,4,5,6,7,8,9] and subsequent return to shelter following failed adoptions [6,9,10,11,12]. In the UK, for example, problem behaviours have been reported as the overall most common reason for relinquishment (34%) and were also responsible for 55% of returns to shelter at one national rehoming charity [4].

Problem behaviours are thought to contribute to the relinquishment risk through many factors, including due to the nature of the problem behaviour such as the risks posed (for example, risk to people from aggressive behaviours, particularly when there are children in the home) [6,9,10,13], the increased care demands (such as time and financial implications for training) [5,8,11,14], care giver burden [14,15] and burnout [16], which can all negatively impact the human–animal bond [2,3,14] and satisfaction with the pet [2,3,11]. Various pet-related factors have also been identified as risk factors in relinquishment, with younger pets [4,6,7] and more recently acquired pets being at higher risk of relinquishment [4,6,12].

Through the extensive research that has begun in this field, the potential role of behavioural advice in preventing relinquishment has become apparent, as there has been shown to be correlations of reduced reports of problem behaviours if pets have been trained [7,8,12], reduced relinquishment if the owner receives helpful behavioural advice [7,9,12] and a reduced number of pets returned to shelters through post-adoption behavioural support [10,11,12,17].

For the individual pet, problem behaviours not only increase the risk of relinquishment but can also negatively impact life expectancy [10,12,15,18] and have detrimental effects on health, such as skin issues [18]. Certain problem behaviours have been identified as particularly important [4,6,10,13,15]. For example, aggressive behaviours have been identified in many studies as leading to a higher risk of relinquishment [4,5,6,7,9,10,13], failed adoptions [5,6,10,12] and higher care giver burden for owners [14,15]. In addition to this, aggressive behaviours can be particularly challenging for behaviourists, as they can outwardly present in similar ways (e.g., a dog biting) but arise from a range of different causes (such as due to fear or frustration) [19,20,21,22], which require different treatment interventions to address them most effectively [9,10]. If the underlying cause is misdiagnosed, there may be a risk that the advice given will not be effective, leading to a risk of relinquishment [7,10]. Unhelpful advice has been identified as a risk factor for relinquishment [7] and could potentially increase the care giver burden (as clients may not enjoy training their pet if it has problem behaviours [13], and training can increase the time and financial demands [7,8,14]). However, if behavioural advice helps the owners, this can reduce relinquishment, as helpful advice has been shown to be associated with pets remaining in the home or not being relinquished [7,9,13]. Therefore, clinical animal behaviourists (CABs) and their advice given can play a vital role in supporting owners to keep their pets [7,9,12], reduce relinquishment [7,9,10,12] and improve the quality of life of both the pets and people they come in contact with [1,14].

Despite the fundamental philosophical importance of the process of behaviour assessment to the field of clinical animal behaviour, this issue has surprisingly received comparatively little academic consideration considering its importance [23,24]. This study aims to explore the currently unknown process by which behaviourists conceptualise, recognise and differentiate emotional states, using fear and frustration as exemplars, given they are commonly involved in problem behaviours that lead to pets being relinquished. Despite the different proposed systems for classifying problem behaviour [25,26,27,28], to date, there is still a lack of international consensus on nosological processes (processes to be followed for the classification of behaviour problems). However, there is growing recognition of the importance of the assessment of emotional basis to problem behaviours, regardless of the final system used for classifying cases [29]. Unfortunately, different terminology is often used by different clinicians to describe both their behavioural assessment and the related emotional states contributing and/or forming part of the behavioural assessment (see [22]). This is not a problem unique to this field, with the description of human emotions clearly showing cultural variation despite some universal commonality [30,31]. Assessing the emotional state of another is potentially challenging, as the state cannot be directly measured, only inferred from related observations in non-verbal individuals [29,30]. Without agreement on a replicable systematic framework that can be scientifically justified, there is a risk of subjective bias going undetected in such assessments, leading to incorrect behavioural assessments [32,33] and a breakdown in communication with both owners and between clinicians and/or researchers. This has important practical implications, as it means that the behavioural assessments cannot be verified or properly evaluated by another, which seriously limits the scientific integrity of the field of clinical animal behaviour. Behavioural assessment also forms the basis for the creation of an appropriate treatment plan [34,35], and so there are wide-reaching practical and welfare implications to how a behavioural assessment is derived. Finally, failure to infer emotions in a consistent way may result in the same term being used to describe different states, both at a clinical/academic level and when discussing assessment with owners.

Fear and frustration are two emotions believed to frequently contribute to problem behavioural cases in practice and may present at least superficially in similar ways [35]. They therefore potentially present a significant behavioural assessment challenge for the CAB. Although these different emotional states may result within the context of a single behavioural problem for an owner (e.g., aggressive behaviour), they require different treatment strategies to resolve the issue in the most efficient, welfare-friendly way, since they represent responses to stimuli perceived in different ways. For example, a dog walked on a lead can bark and lunge towards another dog due to fear (as it perceives the dog as a threat) or due to frustration (as the lead prevents the dogs interacting and thus produces an aversive association) and potentially a combination of both (an animal who cannot find safety when scared may also be frustrated, and an animal that is frustrated may be punished and therefore also become scared). From a clinical evaluation perspective, it can be helpful to focus on one or other as the trigger (or basis) of the initial response, regardless of which emotions might ensue. In the case of a fear-based response to other dogs, the ultimate goal will be to teach the dog that conspecifics are not a threat through a combination of desensitization to the fearful stimulus and counterconditioning a more positive emotional state, whereas in the case of a frustration-based one, the primary goal should focus on establishing a more appropriate way to interact before managing arousal levels [36].

CABs use a range of information to help inform their behavioural assessments and the inference of emotional state. These include the context in which the behaviour occurs, the level of arousal shown by the individual, the behaviour of the individual and the signals it uses [25,28]; these represent objectively describable elements of the components of emotion (proposed by [37]). How this information on different components might be used (or not) by an individual clinician to determine the inferred emotional state of the patient remains largely unknown. In other fields (such as human and veterinary medicine), the decision-making processes, their inherent benefits and their risks have been discussed, and the use of intuition and personal judgement without assessment frameworks have been shown to impact error rates and affect patient outcomes [33,38,39]. For example, a described issue is the risk of making a rapid diagnosis based on intuition alone, as this can lead to errors and an inability to recognise these errors due to the inherent emotional component associated with such decision-making processes [33]. However, the potential benefits of intuition have also been recognised, such as general practitioners sensing when something is ‘not right’ and requiring more thought or intervention, based on gut feelings [39]. Although this area has received interest in other fields, it appears largely unexplored in the behaviour field, and it is unknown if behaviourists use intuition or personal judgement or proposed assessment frameworks when making their assessment. The process by which behaviourists make their assessment could have significant impacts on the risk of a dog being relinquished (for example, if ineffective treatment recommended due to an incorrect behavioural assessment, leading to the owner relinquishing the pet due to the on-going problem behaviour, and a lack of improvement despite their efforts to address the problem). Qualitative research methodologies provide a useful approach to exploring phenomenological issues such as how individuals construct their realities, including their perception of the emotional state of another [40]. Semi-structured interviews can be used to gain understanding and insight into people’s views and enable flexibility to explore topics that may arise during the interviews, making them a useful first step in the exploratory phase of research methodologies [41]. Therefore, this study aimed to examine if and how CABs conceptualise, recognise and differentiate fear and frustration in dogs. Specifically, this study aimed to address the following research questions:

RQ1: What information do clinical animal behaviourists identify and use in order to construct a perception of fear and frustration in dogs?

RQ2: What processes are involved in how clinical animal behaviourists use this information to differentiate fear from frustration in practice?

## 2. Materials and Methods

### 2.1. Ethical Approval

This study was approved by the University of Lincoln Ethics Committee (ethical approval reference number 2021_6675). Electronic informed consent was obtained from participants prior to their participation and was confirmed verbally at the start of each interview.

### 2.2. Participants

CABs are not a regulated profession in the UK as this is not a protected title, so the eligibility criteria of full membership to FABC (Fellowship of Animal Behaviour Clinicians) was used to establish the criterion for the minimum level of relevant knowledge and experience of participants. This allowed for inclusive recruitment from a non-regulated profession. Participants were recruited via email and advertisement on FABC social media (Facebook, Meta, Version 320). Respondents were invited to participate, and ten participants volunteered. These included both veterinary and non-veterinary behaviourists. Subjects are labelled A–J for reference when providing direct quotes.

### 2.3. Interviews

A standard semi-structured interview format was created (Supplementary Material Script S1) and pilot interviews conducted. All interviews began with gathering some basic demographics concerning the respondent and then moved to a series of interview questions based around three topics; recognition of fear, recognition of frustration and how these emotions are differentiated. In relation to the recognition of fear and frustration, the topics discussed included definitions, behavioural tendencies, communicative signals and terminology used to describe these emotions when discussing cases both with clients and veterinary surgeons. Semi-structured interviews were used to ensure all participants were asked the same fundamental questions in order to ensure consistency across the interviews. In order to minimise the impact of interviewer bias, the semi-structured questions were assessed by the other authors, which included a non-veterinarian (CS) and a veterinary behaviour specialist (DM) to ensure the questions were inclusive and comprehensive.

Half of the participants were asked questions relating to fear initially, and half of the participants were asked about frustration first, in order to counterbalance any information-related order effects.

Semi-structured interviews were conducted by the first author (BW), and interviewer bias was minimised by following the pre-defined set format of the semi-structured interviews and aiming to avoid any comments in response to answers provided. Open-ended questions were used initially in order to avoid leading or influencing the participants, followed by closed questions as required. Any emergent topics arising from exploring the depth and breadth of the topic were included in all subsequent interviews with the remaining participants until redundancy was achieved.

The interviews initially transcribed automatically using Microsoft Teams (Microsoft Corp, Version 24215.1007.3082.1590.) into Microsoft Word (Version 2408) before reviewing and manual editing for accuracy, with grammatical interjections removed. Interview duration varied from 29 min to 90 min (mean ± SD = 51 ± 22 min). Participants were recruited purposively with a view to obtaining diversity within the available population and continued until no further volunteers were available using the agreed recruitment strategy. Although such qualitative research inherently has a small number of participants, it ensures the topic is explored by breadth and depth to gain an understanding of potential issues and topics that require further exploration through other means in the future.

### 2.4. Analysis

A post-processing structure was imposed on the transcriptions to group the data into three sets: one relating to fear within RQ1, one relating to frustration within RQ1 and one relating to the differentiation of the two emotions (for RQ2). These data were then imported into the NVivo software (v12) for emergent coding and analysis. This was achieved through an iterative process of identifying topics present and coding statements related to these topics. Through an iterative process, all the interviews were re-examined and coded until no further codes were identified. These codes were then reviewed, and any overlapping codes (which contained information on the same topic but labelled with different terms/codes) were merged (into one common code that encompassed all previously separately coded topics) to generate a more rigorous structure.

Thematic analysis was conducted, and overarching themes were identified from the coded data by grouping the topics into common themes/topics. These themes were reviewed and reclassified in an iterative process and reviewed independently by all authors until the final themes were decided upon. Codes and themes were checked for validity by reviewing random codes and themes to ensure they had been appropriately labelled and identified. The information relevant to the two research questions was then extracted as detailed below in Table 1.

## 3. Results

All participants had significant experience with seeing dog cases as CABs and had achieved qualifications in animal behaviour, ranging from Postgraduate Diploma to PhD (Supplementary Material Table S1). Since our interest was in the qualitative rather than quantitative aspects of the responses, the dataset was analysed as a whole and not stratified by factors such as level of education.

### 3.1. General Views of Fear and Frustration in Relation to Problem Behaviour

Fear and frustration were broadly recognised as forms of emotional reaction by the interviewees, with terms such as “*affect*”, “*valence*” and “*feeling*” often used in reference to these states. Affect is the state of experiencing feelings and encompasses emotions and moods [42], whereas valence can be thought of as the intrinsic emotional value ranging from positive to negative [43].

All the interviewees indicated that these states bring about general changes in the animal’s behaviour in response to certain stimuli or the animal’s perception/appraisal of these stimuli, with some highlighting the physiological basis for this. For example, E’s definition of fear was:

“*…fight or flight system …, because … something unfamiliar, scary, loud or whatever has triggered a physiological response in the dog*….”

Fear and frustration were often described as overlapping states that co-occur with problem behaviour, rather than occurring in isolation, and this can make distinction challenging, e.g., F:

“*it’s such a difficult thing because … we tend to … give names to these kind of emotions and describe them but actually it’s very difficult because I think they are not discrete emotion. How … can you divide fear from frustration for example? Because if they cannot cope in keeping the threat away and they become frustrated …, … so it’s difficult*”

Although interviewees tended to reference fear and frustration as emotional reactions, there were some references to their relationship with aspects of temperament and personality, suggesting that they are also perceived as a more general predispositional state. It was recognized these states contribute to behavioural individuality, e.g., D:

“*… I would say the expression of an emotion is very individual thing*.”

The interviewees each gave diverse definitions of fear and frustration, suggesting there is no consensus definition used for either state. Many interviewees did not define fear or frustration in a way that could be considered operational, and many used a degree of tautology in their definitions, e.g., describing fear as a response to something scary.

The functional value of these emotional states was alluded to by several respondents, e.g., F referred to fear as “*an adaptive kind of response*”, with some emphasising this role in relation to dealing with stressors and the potential for them to appear maladaptive in cases of problem behaviour. For example, in relation to fear, I said it is:

“*a normal stress response, but it becomes maladaptive when welfare is compromised and when they are experiencing exposure to perceived triggers of fear regularly*.”

Both fear and frustration were described by some as potentially leading to a loss of the functionality of the behaviour, e.g., F:

“*If you look … at the dog that is trying to prey the squirrel in a normal way, you might see … staring, slow movement and so on, and then …where the squirrel is able to get to the tree that might get frustrated …they started to lose all the functionality of their behaviour and just bark jump, barking, jump. And this is something that is not functional. Just an explanation of their emotional state*.”

### 3.2. RQ1 “What Information Do Clinical Animal Behaviourists Identify and Use in Order to Construct a Perception of Fear and Frustration in Dogs?”

The extent to which the interviewees had similar or different theoretical perspectives for each of these emotions is described in separate subsections below.

#### 3.2.1. Fear

Fear was broadly recognised as a negative affective state with the goal of avoidance of a potentially harmful/painful stimulus, with responses motivated by self-preservation, e.g., A:

“*so an emotional response based around, mainly self-preservation… it’s negative emotional response towards the stimuli that could potentially cause harm*.”

The interviewees tended to refer to fear occurring in the presence of a threatening stimulus, with some interviewees wanting to differentiate fear and anxiety on the basis of the predictability versus presence of the threat, e.g., J: “*fear is the experience of, where anxiety is the anticipation of …*”. In addition, some also referenced a relationship between panic and fear, e.g., G: “*Again there is a fine line between when fear becomes a panic*”, whereas other interviewees referred to anxiety and fear co-existing, e.g., E:

“*fear is when you want to get away from something, whereas anxiety is where you worrying about it’s going to be there… so you’re worried you’re anticipating something being fearful, so you’re effectively fearful in the moment, but it’s anticipatory fear. So no different things, but you do get you know this commingled thing on occasion*.”

A wide range of possible observable behaviours/communicative signals were described by several interviewees. It was generally acknowledged by many interviewees that behaviours observed were not unique to fear, as these could also be attributed to “*acute stress*” (F) or “*frustration*” (B) for example. F’s description was representative of many participants: “*there is not a really precise fingerprint for fear*”. Nonetheless, some alluded to “*classic*” behavioural signs associated with fear, e.g., F described such signs as “*low body posture, wide eye, ears backwards, tail behind the legs*”. By contrast, some considered that almost any behaviour might reflect fear and made reference to the function of the communicative signals, e.g., D:

“*…it’s so widely ranging, I think that it’s almost every single behaviour that an animal can display … in response to fear, so I just don’t think you can even parcel it. You can have appeasement based behaviours,… play based behaviours,… avoidance based behaviours…, antagonistic based behaviour, so … I couldn’t pigeonhole it. I think any single behaviour could be indicative of fear*.”

On the other hand, other interviewees said they identified fear using body language, e.g., E:

“*It’s just clear fear and in some cases it is just clear frustration and you’d have to base that around body language*.”

The interviewees often referred to aggressive behaviours occurring when a dog was fearful; however, they referred to diverse reasons for this. For example, C referred to “*an overlap there with frustration*”, whereas H attributed it to when “*there’s been more rehearsal of that behaviour*”, whereas E referred to reaching a threshold:

“*below threshold would choose to avoid, but once over threshold might choose to go in and fight for it*.”

Throughout the interviews, frequent reference was made to associated behavioural motivations, e.g., H described aggressive behaviours as “*fear defensive type*”, whereas B suggested “*the animal can be very cowed, trying to move away from the thing that is worrying them*”. Others made reference to “*personality*”, such as D: “*a bolder individual may use antagonistic behaviours*”.

Contradictions were common, both within and between interviewees’ responses, for example, as demonstrated in the above quotes regarding communicative signals expected in fear.

#### 3.2.2. Frustration

Frustration was also broadly recognised as a negative affective state, with the interviewees frequently making reference to concepts such as “*anger*” (D, E and F) and “*disappointment*” (J). These comments were often complemented by reference to limits to the animal’s autonomy, e.g., A defined frustration as:

“*a response to a stimuli (sic) where either the animal has been prevented from achieving a goal or wants to keep hold of something, that is important to them creating an invigoration of behaviour towards further attaining that goal*.”

A range of behavioural manifestations of frustration were described by the interviewees, including “*over aroused*” (B), “*repetitive*” (D), “*cross*” (E, F), “*escalating*” (H), “*persistent*” (J) or the dog can be “*grabbing, mouthing, ragging, … redirecting*” (G).

Attempting to access or approach the stimulus was often described in relation to frustration, e.g., H:

“*more forwards than backwards,… trying to interact in some way with that stimulus, whether it be visually or from a… mouthing type perspective, or whether it’s just… whole body closeness*”

Some interviewees made reference to frustration co-occurring with other emotional states, e.g., B:

D: “*it’s a very strong negative emotional state again depending on arousal, especially if the frustration and the goal is tied up with avoidance, so they don’t necessarily occur on their own, so if the goal is I want to move away and you’re getting frustration based on the inability to move away then you could certainly have all of these things going on at the same time*.”

A wide range of possible behaviours was described by several interviewees. It was acknowledged these behaviours can be “*high intensity*”, “*repetitive*”, “*difficult to redirect*” and not necessarily directed at the goal, e.g., D:

“*it’s not goal directed at the goal because if they know that they’re being thwarted from the goal, they can just push it anywhere*”

However, many interviewees referenced the lack of unique signs of frustration, e.g., J:

“*I would say that there probably isn’t specific behaviours that are indicative of frustration*”

Reference was made to behaviours being “perceived as … fear or anxiety” (I), “stress” (G) or “panic” (F), whereas others alluded to these signs changing (C): “I think some individuals will give up quickly if they become frustrated and even start showing signs of anxiety and fear”.

Some interviewees referred to “*self-directed behaviours… repetitive behaviour, self-injurious behaviour*” (F) occurring in frustration. It was generally acknowledged that aggressive behaviours can occur in relation to frustration:

A: “ *whining, barking, lunging, pulling, trying to decrease distance, padding of their feet so restlessness. Sometimes growling, snapping, biting or grabbing as well*.”

In general, the interviewees gave briefer descriptions and less information when discussing frustration compared to fear, with frequent references to the difficulty in identifying frustration, often using their assessment of context and motivation, e.g., B:

“*it would depend if there’s some social aspect, may be that the dog is trying to communicate like if it really wants to get to another conspecific or their favourite person is the other side of the gate and they can’t reach them and they’ve just come through the door*.”

### 3.3. How the Presence of a Given Emotional State Was Determined in Practice

The interviewees frequently made reference to the difficulty in distinguishing fear and frustration, but many emphasised the importance of body language and context, e.g., E:

“*… sometimes it is actually quite difficult, but you would be looking at the body language…, the choice and…, when things occurred and how they occurred and what the focus of the attack was*.”

The interviewees referred to the following areas of importance in relation to each emotion, which can be largely mapped onto the component process model of emotion [30,37] (Table 2):

### 3.4. How Respondents Applied Their Theoretical Understanding in Practice, and to What Degree This Was a Scientifically Rigorous Process

The interviewees referred to motivations and feelings to justify the communicative signals described, and confirmation bias was often present, e.g., F:

“*if the dog want to avoid it might try to keep …as much distance as possible. But if this animal learned that this is not effective might still… have this fight reaction*”

Other participants referred to the lack of distinguishing communicative signals (which directly contradicts other interviewees, as described above), e.g., B, when asked about which features must be present to make the distinctions of fear and frustration, referred to:

“*The stimulus that’s present, its relationship with that stimulus, its history but …the body language can often be very similar*”

Tautology was a frequently noted feature of the interviews. However, there was reference to evidence falsification by some interviewees, e.g., I: “*wouldn’t usually expect it in a fear response*”, whereas others referred to intuition, e.g., J:

“*so I think it’s really difficult because… I think …I feel it and know it, rather than…, I do read it, but I think it’s almost an automatic oh that dog is frustrated or mostly frustration.*”

### 3.5. RQ 2: “What Processes Are Involved in How Clinical Animal Behaviourists Use This Information to Differentiate Fear from Frustration in Practice?”

#### 3.5.1. The Perceived Importance of Differentiating These States in Practice

Most interviewees implied they distinguish these emotions, and some interviewees made reference to the challenges of differentiating fear and frustration (see above). Some interviewees provided an explanation for the reported difficulty, and these included the following:

F: “*this discreet definition of emotion doesn’t fit in my experience honestly. And what you see in an animal is something that is often the result of different kind of… experience it had in the past and different …way of …putting together all the strategy, that it had in the past and so on and sometime is really difficult to distinguish these two emotions, but of course, if you have clear expression of fear we’ve a lot of these autonomic signs*”

E: “*quite difficult, but you would be looking at the body language and …at the choice and …what, when things occurred and how they occurred and what the focus of the attack was*.”

Other interviewees reported they may not distinguish the emotions, e.g., C:

“*I wouldn’t necessarily make a distinction between the two. I could say there’s a dog there that’s scared and frustration isn’t playing a huge role at the moment. Or there’s a dog that is frustrated and fear isn’t a big role, but it would just depend on … the context, so the history of the context”…”…but I wouldn’t say distinguish completely between the two*.”

One interviewee (I) also made reference to their concerns about trying to distinguish these emotions:

“*it’s so context specific … that’s why I’m struggling to answer the question, because to me …just giving an immediate label is so, it’s almost a bit dangerous, …I think because we have to we really have to be so aware that we are getting the motivation right so we can approach the treatment appropriately…*”

As can be seen from the quotes above, the interviewees used circular reasoning, confirmation bias, intuition and tautology when differentiating fear and frustration, and many did not have a clear systematic framework they could articulate. The interviewees often referred to emotions co-existing.

#### 3.5.2. How Respondents Distinguish Fear and Frustration, including Features That Must Be Present or Absent to Allow for This Distinction or the Ruling in/out of a Given Emotion

Most interviewees made reference to using a combination of factors when assessing the emotion involved, as summarised by G:

“*…the history, the body language, the context, the sociability of the dog, … how much fear there is?… the ruling out of the medical factors…how the dog is interacted with on a daily basis, what their general personality is so they are fearful or frustrated and …how that dogs been around different situations*.”

Reference was made to collecting evidence with a view to supporting the behavioural assessment of one emotion over another, e.g., C:

“*… I would take all of them basically and then come up with the diagnosis rather than … just basing it on one the observable behaviour for me is super, super important, but it’s not …the be all and end all*”

By contrast, some interviewees appeared to use a more robust process of falsification as part of their process by excluding differentials based on the information obtained:

D “*… once there is the ability for them to attain their goal themselves or attain the ECS themselves if that calms the situation down and there’s a good interaction I would then potentially lean more towards frustration and rule out fear”… “avoidance for me would rule out frustration*”

(ECS refers to Emotionally Competent Stimulus, i.e., a stimulus capable of eliciting an emotional response, but not necessarily doing so.)

E “*if the dogs completely avoiding a situation, then they’re not frustrated*”

F “*I would say a frustrated animal would not tend to avoid but to approach, and a fearful animal will mainly tend to avoid*”

G “*it’s almost like the ruling out of the things*”

As shown through the examples given, some interviewees appeared to contradict themselves during the interview.

### 3.6. The Quality of the Processes Used to Differentiate Fear and Frustration

The interviewees made reference to assessing multiple factors when assessing an animal, including the context, motivation, temperament, style and learning history. However, most interviewees described a process in which they assessed this information to reach a behavioural assessment and did not refer to using a structured/formal or consistent process. The interviewees also made reference to emotions occurring concurrently or on a scale, rather than as discrete entities, for example, C’s response to differentiating fear and frustration was:

“*I don’t I guess you could say. I …have them both in my mind and think they just display in different degrees with each other so …definitely have a fearfully and frustrated dog… I’ve got …fear on one side and then maybe … seeking something nice on the other side and frustration is in the middle, overlapping both of them*.”

## 4. Discussion

This study established the difficulties experienced by CABs in differentiating fear and frustration when assessing problem behaviour in pets. We showed that there is diversity in the perceptions of fear and frustration among CABs, and this could have implications for the behavioural assessment reached and, as such, may impact the effectiveness of advice given and subsequent relinquishment risk [7,9,13]. This is of clinical importance, as different assessments should lead to different types of intervention if treatment is to be specific and of most use to the owner [7]. For example, the selection of psychoactive medications may change depending on the emotional states considered to make up the diagnosis by the veterinary behaviourist [44]. As such, our results highlight the challenges associated with the practical application of this widely used terminology by clinicians, academics and owners. Each behaviourist appeared to have their own personal construct of fear and frustration. This is perhaps to be expected given the lack of a consensus definition of emotion, let alone specific states within it [30]. There have been a range of proposals aimed at providing clarity to the definition and concept of emotions, such as a focus on the use of scientific versus colloquial definitions [45], and the merits and constraints of different scientific definitions have been discussed elsewhere [30,46,47,48]. Given the diversity of views concerning fear and frustration, it is imperative for researchers and behaviourists to define such terms when reporting on them to others in order to establish what they mean, rather than leaving it to the reader’s intuition. Likewise with clients and members of the public, a clear explanation in lay terms of what is meant when using these terms is likely to be helpful, since they too may have their own definitions, although this remains to be established.

The respondents expressed a range of beliefs concerning the temporal nature of fear and frustration (from emotional responses to temperament traits), their related stimulus properties (including some participants distinguishing fear and anxiety based on the presence of specific stimuli), the associated responses (such as communicative signals) and related constructs (such as emotions co-occurring). Within the literature, anxiety can be used to describe the anticipation of a fearful event in relation to motivational conflict (which is believed to be more closely associated with frustration and a loss of autonomy) [49] and in relation to repetitive behaviours due to frustration [50]; however, fear and anxiety can also be considered as part of the same affective system [51]. Anxiety and fear can be considered challenging to clinically differentiate and are often differentiated based on the assessment of the animal’s response and the perceived trigger [19,21]. Despite these recognised issues within the field, there was consensus among the CABs on some aspects, for example, that these emotions were of negative valence. Emotions can be assessed in many ways, including primarily in relation to (dis)pleasure and valence [30,52]. Another approach focuses on the definition of essential components underpinning an emotional response, e.g., context, arousal, action tendencies, communicative signals and subjective feelings, in order to define specific types of emotion [30]. The behaviourists in this study made reference to these elements that underpin the component process theory [30,37].

The CABs also made reference to these emotions co-occurring and this adding to the challenge of making a behavioural assessment (despite apparently using multiple factors such as component process theory to triangulate and differentiate the emotions involved to some extent). This difficulty could in part be due to them both being of negative valence or of similar valence and arousal [53] and therefore potentially more difficult to specify, compared to a more general contrast in valence, i.e., a negative versus positive valence state [54,55]. However, it could also be, in part, due to the apparent similarity in observable signs (including body language, behavioural tendencies and, in some cases, contexts) between fear and frustration [20], which further complicates the behavioural assessment and differentiation of the emotions. Given the importance of owners receiving helpful behavioural advice [7,9,13], this issue will be explored further in future studies (by the authors) to assess if there are processes to aid the differentiation and ultimately the effectiveness of the behavioural assessment and subsequent advice. However, the results highlight the value of adopting a systematic process for inferring emotion, such as that proposed by Mills [8], which uses the components of Scherer [30,37] as lines of evidence in an inductive reasoning process, and the authors propose that such methods are offered here as potential solutions in the short term.

Different methods of assessing problem behaviours have been proposed [23,29], and to the authors’ knowledge, the current work is a novel exploration of how those working in the field are applying the assessment of emotion, which is integral to the process of problem behaviour assessment [21,25,44], in practice. Many participants appeared to use intuition and gathering evidence in support of a proposed assessment when attempting to reach a behavioural assessment, with evidence of tautology and circular reasoning supporting an approach grounded in confirmation bias. However, some participants referred to falsification in line with scientific principles, but this was not common, and several participants often appeared to struggle to apply the principles of falsification when challenged about this in later parts of the interviews. The use of intuition is not unique to the field of clinical animal behaviour and can be beneficial in some circumstances; see [39] for an example from the human medical field. However, the use of such decision-making processes without a structured, scientific framework in behavioural assessment inference could increase the risk of confirmation bias and reaching the ‘wrong’ conclusions (akin to misdiagnosis) [33]. In other fields, the use of decision-making frameworks centred around the principles of avoiding dependence solely on confirmation bias and intuition has shown promising signs in reducing the risk of errors and increasing the scientific rigour and reliability of diagnoses [33,38,39]. This is therefore an important topic for further consideration within the animal behaviour field.

The aim of this type of qualitative work is to explore in detail potential issues of concern rather than to quantify them. However, given that the participants were all members of a respectable practitioner organisation with relevant independent qualifications and experience, it seems reasonable to argue that the issues raised are probably a general issue. As the terms “behaviourist” and “clinical animal behaviourist” are not protected titles, some working in this field may have little or no scientific training or insight; we would argue that greater diversity in the behavioural assessment process, vocabulary and definitions probably exist than is suggested here. This work highlights not only the importance of defining key constructs such as those relating to emotional state but also the need to agree replicable scientific frameworks for determining these states and decision-making frameworks [33,38].

The scientific reporting of nosological issues within the field of clinical animal behaviour appears to be a largely neglected field of study. To date, there are three main overarching frameworks described to address this nosological challenge within the context of problem behaviour in companion animals: the approaches described by Overall [26], the approach described by Pageat [27] and the approach described by Mills [29]. Both the approaches of Overall [26] and Pageat [27] take a medical approach to behavioural problems. Concerns over the medicalisation of problem behaviour have been reviewed previously (e.g., [25,56]). Both of these adopt the common medical approach of necessary and sufficient criteria in order to define specific disorders or pathological states, although this is perhaps much more explicit in Overall’s definition [26]. By contrast, the process described by Mills [29,32], described as a psychobiological approach, emphasises the importance of assessing normal functional motivational and emotional processes that are on-going in the animal, with an emphasis on falsification of inferences relating to these internal states [28,32]. It is worth noting that in our current dataset, no reference was made to pathological processes. This may reflect a geographic bias, with the medical approach not thought to be widely adopted in the UK, or it could be the product of sampling limitations, although we did not directly explore which nosological system(s) the participant reported to use. It may be possible that different approaches are used in other countries based on the predominant approach of the country the clinician is practicing in or based upon the training they received and subsequent continuing professional development. Clearly, this is an area which deserves further scientific attention. The issues regarding conceptualisation, semantics and consensus definitions are not unique to the field of clinical animal behaviour but are of fundamental importance with extensive debate on the nature of fear [56].

## 5. Conclusions

These results show the challenges reported by behaviourists in differentiating emotions (such as fear and frustration) and making a behavioural assessment. While there was general agreement in some concepts, such as the relationship between fear and threatening stimuli, the process involved in differentiating between key emotional states was much less consistent, and challenges experienced were articulated. Having greater precision in and articulation of the terminology used alongside a description of the processes used to make an inference are useful first steps. With time, it can be hoped that there might be greater development and adoption of rational frameworks to allow, at least, clinicians and academics to communicate more effectively and ultimately improve problem behaviour for pets and owners to improve welfare and reduce relinquishment. A precondition of this is undoubtedly greater awareness of the issues identified in the work reported here.

## Figures and Tables

**Table 1:** The information used from the interviews to address the research questions.

Research Question to Be Addressed:	Responses Used from Interviews Relating to the Following Elements of the Interview:	To Allow for Assessment of:
RQ1 “What information do clinical animal behaviourists identify and use in order to construct a perception of fear and frustration in dogs?”	“What is your definition and understanding of fear/frustration in dogs?”	To what extent respondents had similar or different theoretical perspectives for each of these emotions
	Questions relating to the behaviours used to indicate each emotional state and how they would recognise this state in a dog	How the presence of a given emotional state was determined in practice How respondents applied their theoretical understanding in practice and to what degree this was a scientifically rigorous process
RQ 2: “What processes are involved in how clinical animal behaviourists use this information to differentiate fear from frustration in practice?”	How they distinguish fear and frustration The features that must be present or absent to allow for this distinction and which features allow for each emotion to be ruled in or out of a case	The perceived importance of differentiating these states in practice and the quality of the processes used to achieve this

**Table 2:** Examples of responses given by interviewees in relation to each emotion with mapping onto the component process model of emotion.

	Fear	Frustration
Context, including triggers	Appraisal D: “*as an animal knows how to deal with the fear and can cope with it*” Stimulus properties H: “*stimulus whatever that might be … it might be noise… visual, … be a mixture of all of those*” E: “*something unfamiliar, scary, loud*”	Appraisal C: “*physical barrier or a psychological barrier*” F: *“lack of proper stimulation, restricted environment, deprivation of exercise*” I: “*context for frustration, like whether a dog is tethered and doesn’t want to be*” Stimulus properties B: “*I guess or whether their activity is directed at a particular barrier or some other sort of restriction like the lead, garden fence…*” I: “*desired object or social stimulus*” I: *“there’s a ball, it’s rolled under a sofa and they’ve tried to get it and they can’t*”
Behavioural responses, including signals	Behavioural tendencies B: “…*looking away. Unless it’s staring at something and they’re trying to go on the offensive to repel.*” E: “*below threshold would choose to avoid, but once over threshold might choose to go in and fight for it*” F: “*express the classical biological responses like fight, flight, freeze…*” Communicative signals C: “*… low body posture, moving away from, things that might be umbrellaed of appeasement- …ears back, looking away, moving away, ducking away, hiding.*” E: “*Often to the backward leaning body posture, because I think that’s quite good one to be indicative of fear and it helps them with their sort of confusion about fear and frustration*.” Escalation in signs G: *“this sort of milder lip licking, the ears back, tail low, rolling on the back, this sort of appeasement grin, escalating all the way up to the more aggression, the more overt aggressive behaviour*.” Avoidance D: “*given enough space there will be avoidance*”	Behavioural tendencies C: “*whether they kind of give up and get a bit sad or whether they try harder and get more invigorated*” D: “*higher intensity, repetitive and depending on what we’re goal is sometimes difficult to interrupt*” Communicative signals G: “*very breed dependent*” G: “*higher pitch for the sort of frustrated, pro social bark*” H: “*I see that with the vocalizations …they become more intense the more frustrated the animal gets. …often starting off low level and then seeing that ramping up really.*” H: *“quite like repetitive movement patterns, sometimes escalating movement patterns for example if they’re trying to access something up or get out of something that they want to be away from that you might see kind of an escalation in the intensity of that behaviour*” I: “*physical agitation*” C: “*whole range depending on the cause of frustration and how physiologically aroused the dog is at the time*”
Signs of arousal	D: “*in a fear, like a solid fear is maybe elevation of like things that are indicative of arousal, elevation of heart rate, dilation of pupils, heart rate*” D: “*high arousal state of fear, then they would be raised, but not if there are low arousal state of fear but you could also get the hackles raised for different emotional states as well*” B: “*physiological so there would there would be highly stressed dogs physiologically*.”	F: “*Well if you imagine that this dog is really jumping towards barking, and so on, so tense body… he might have increased respiratory rate, but not at the level of this scared animal. You will have an animal that is more control, it’s not…panicking like, …losing control of sphincters or things like that*” G: “*I think the arousal levels would generally be quite high, in my experience, may tend to be higher, but that’s with any emotional response*” B: “*over arousal at not being able to get the thing they want*” D: “*I would say it can be very low arousal. It can be a low arousal level of communication where they’re just trying to say I want this. Or it can be very high level arousal, so all the way through the spectrum*”
Other	Motivation A: “*just trying to get away as well so just really trying to increase distance*” Learning history G: “*how the development of the fear behaviours has got, so whether it’s the dog feeling fearful for the first time they’ve encountered that stimulus, or if they are well practiced at*.” *General assessment* F: “*in what context the animal expresses, it really depends on the individual, so temperament situation in which the animal is” * Feelings C: *“If the dog is scared unless it is really panicking and caught for me it’s more of it be quiet but if they are panicking and caught, then like this shriek, scream type behaviour, you more likely to hear …for a dog that’s really scared*” D: “*A feeling of vulnerability*” Justification/inference to motivation B: “*Tucked, lack of movement. Unless there’s stiff and staring at the thing that’s worrying them and trying to repel them.”* F: “*If it’s really terrified and he’s not able to communicate, you might just see that it’s frozen.*” Variation C: “*there could be quite a lot of variation depending on their the thing that’s making them scared and the dogs learnt history*” *Breed and genetics* G: “*breed and all of that’s going to and genetics potentially are going to have an impact on all of these*” Classification H: “*defensive fear related behaviour”, “territorial based fear*” Style: H: “*really fearful of other dogs approaching and becomes more kind of defensive, aggressive*” I: “*pulled back, fearful ears*”	Motivation D: “*there is normally a goal in mind… you should have observed kind of a movement towards the goal or attempt to attain the goal or desire initially.*” D: “*… especially if the frustration and the goal is tied up with avoidance, so they don’t necessarily occur on their own, so if the goal is I want to move away and you’re getting frustration based on the inability to move away then you could certainly have all of these things going on at the same time.*” Dynamism H: “*whilst the dog is still in that kind of active frustration phase …once they …start to shut down, if that is the sadly is the case then that obviously changes but I think was active so frustration*” G: “*You might see a more impulsive individual so more forward, trying to act on its motivation*.” Temperament J: “*frustrated dogs tend to show frustration in lots of different contexts as well*” F: “*frustration and impulsivity run together*” Other emotions F: “*different kind of expression of frustration, possibly depending on the individual features, both genetic and learned*” Style: A: “*Intense*” B: “*hyper, lots of movement*” B: “*over aroused*” I: “*there’s something about the … physical agitation…*” I: “*agitated, but purposeful*”

## Data Availability

Anonymised data are only available on request from the corresponding author (bewilson@lincoln.ac.uk) due to confidentiality and privacy obligations to the participants.

## Supplementary Materials

The following supporting information can be downloaded at: https://www.mdpi.com/article/10.3390/ani14182718/s1, Supplementary Material Script S1: Script for semi-structured interviews, Supplementary Material Table S1: Participants qualifications and experience in the field.
