# Supplementary material for: Problem Behaviours and Relinquishment: Challenges Faced by Clinical Animal Behaviourists When Assessing Fear and Frustration
Source: Animals (Basel). 2024 Sep 19;14(18):2718. doi: 10.3390/ani14182718 (PMC11428939; doi:10.3390/ani14182718)
Supplement: Supplementary file 1 [file animals-14-02718-s001.zip › animals-3192600-supplementary.pdf]

Supplementary Material Script S1

Script for semi-structured interviews

Stage 1: Introduction to experiment

Have you read and understood the participant information sheet?
Do you consent to participate in this study and for this session to be recorded?
Are you an animal behaviourist?
Which professional organisations are you a member of?
Would you like to ask any questions at this stage?
If you are happy to continue you will now be asked some questions about dogs.

Stage 2: Experience

Please can you give me a brief overview of your experience in the field of animal behaviour?
Are you a vet or veterinary nurse?
What behaviour qualifications do you have?
How long have you worked with animals?
How many years have you worked as a behaviourist?
Approximately what percentage of your work as a behaviourist is with dogs?
Do you or have you ever owned a dog? If so how many years have you owned a dog?
Is there anything else you would like to add about your experience?

Stage 3- FEAR (the order of FEAR and FRUSTRATION were alternated as detailed in the methods section)

What is your definition and understanding of FEAR in dogs?

For the following please describe what you view as FEAR, applicable to all situations when an animal is fearful, rather than one specific context/example. i.e: something that is not just relevant to noise fears.

What behaviours do you consider indicative of fear?
How would you describe a fearful animal?
Example if needed: the way a dog looks when running for a treat, rather than running after prey?

Style of behaviour
E.g. the way the dog does something
Specific example if needed: running for a treat.
Whole body movement
Body posture/the way the dog carries itself
Muscle tone
Distance the dog chooses to be from the stimulus
E.g. does the dog stay where they are, approach or move away
Specific example if needed: dog treat thrown on floor
Is there anything else you would like to add?
Again please describe for FEAR applicable in all situations
Head
Position. Example: leaning forward, back, neutral, tilted
Ears
Example: forward, back, neutral. Stationary or moving
Eyes
Gaze. Example: Pupils, opening, sclera, blinking
Mouth
Example: open, closed, lips, teeth, tongue.
Nose
Example: Licking, sniffing

Forehead
Example: furrowed brow, neutral.
Does this apply across all fearful conditions?
Is there anything else you would like to add?
Other signs
Vocalisations
pitch, intensity, duration, volume. Example: whining, barking, whimpering, yelping
Hackles
Tail
Position, movement + speed
Physiological signs
Example: panting, drooling, heart rate
Does this apply across all fearful conditions?
Is there anything else you would like to add?
What features of the dogs' body language and behaviour do you draw the clients attention to?
What language do you use when speaking to the clients about FEAR?
What descriptive terms do you use to describe a fearful animal in your clinical notes? Letters to owners and vets?
If they are different for owner and vet, why?

Stage 4- FRUSTRATION (the order of FEAR and FRUSTRATION were alternated as detailed in the methods section)

The questions from stage 3 are repeated for frustration.

Stage 5- Distinguishing FEAR and FRUSTRATION

How do you distinguish fear and frustration?
What features must be present for you to make this distinction?
What features must be absent for you to make this distinction?
Which features allow you to rule in/out fear and frustration as a diagnosis?

Stage 6- Wrapping up

Is there anything you would like to add about anything we have discussed?
Thank you for your time.

Supplementary Material Table S2

Participants qualifications and experience in the field

Veterinary surgeon (VS) or nurse (VN)	Qualifications	Time worked with animals	Years worked as behaviourist	Percentage of caseload of dogs	Dog owner?	Time owned dog(s)
No	Masters in Clinical Animal Behaviour	18 years	6	100%	Yes	3 years
No	Bachelors in zoology and psychology, PhD	15 years	6	70%	Yes	30 years
No	BSc in Behaviour and welfare, MSc by research	14 years	10	100	Yes	4 years
No	Undergrad degree in zoology, Masters	7 years	4	99%	yes	7 years
No	Postgraduate diploma (Companion animal behavior counseling)	16 years	14	100%	yes	30 years
Yes, VS	Diploma in advanced companion animal behavior counseling, Masters in companion animal behavior counseling, Masters in companion animal behavior, PhD in animal science, full member of the European College for Animal welfare and behavioural medicine and recognized specialist in clinical animal behavior from the Royal College of Veterinary Surgeon.	34 years	20	100%	Yes	50years
no	Masters	9 years	2	100%	No	n/a
No	Masters	25 years	15	70%	Yes	25 years
No	Undergraduate degree, Masters (animal behaviour)	10 years	5	98%	Yes	7 years
No	BA Hons (companion animal behavior consulting), MSc (companion animal behavior)	19 years	14	100%	Yes	19 years
